# Comparison of the Effects of Two Different Drinks on Microhardness of a Silorane-based Composite Resin

**Published:** 2015-09

**Authors:** Sedighe Sadat HashemiKamangar, Maryam Ghavam, Zhina Mirkhezri, Mohammad Javad Karazifard

**Affiliations:** a Dept. of Operative Dentistry, School of Dentistry, Tehran University of Medical Sciences, International Campus, Tehran, Iran.; b Dental Research Center, Tehran University of Medical Sciences, Tehran, Iran.; c Dentist, Tehran University of Medical Sciences, International Campus, School of Dentistry, Tehran, Iran.; d Dept. of Epidemiology and Biostatistics, Dept. of Public Health, School of Dentistry, Tehran University of Medical Sciences, Tehran, Iran.

**Keywords:** Beverages, Hardness, Silorane Composite Resins

## Abstract

**Statement of the Problem:**

Acidic foods and drinks can erode composite resins. Silorane-based composite is a new low shrinkage composite with higher hydrophobicity which might resist the erosive effect of beverages.

**Purpose:**

The aim of this study was to determine the effects of 100% orange juice and non-alcoholic carbonated beer on microhardness of a silorane-based composite in comparison with two methacrylate-based composite resins.

**Materials and Method:**

Ninety disc-shaped composite specimens were fabricated of Filtek P90, Filtek Z350 XT Enamel and Filtek Z250 (3M-ESPE) (n=30) and randomly divided into 3 subgroups of 10.Group 1 was immersed in distilled water, group 2 in 100% orange juice, and group 3 in non-alcoholic beer for 3 h/day. Primary, secondary and final Vickers microhardness tests were performed at the beginning of the study and 7 and 28 days later. Surface of 2 specimens in each group was evaluated under scanning electron microscope on day 28. Data were analyzed using repeated measures of ANOVA model (α=0.05).

**Results:**

The primary and secondary microhardness of P90 was significantly lower than that of Z350, and Z250 (*p*< 0.001). Microhardness of Z350 was also lower than that of Z250 (*p*= 0.002). On day 28, microhardness of P90 was lower than Z250 and Z350 (*p*< 0.001); however, microhardness values of Z250 and Z350 were not significantly different (*p*= 0.054). Microhardness of specimens subjected to non-alcoholic beer was significantly lower than that of controls (*p*= 0.003). Meanwhile, the microhardness value of resins in orange juice was somewhere between the two mentioned values with no significant difference with any of them (*p*> 0.05).

**Conclusion:**

Although 28 days of immersion in 100% orange juice and non-alcoholic beer decreased the microhardness of all specimens, P90 experienced the greatest reduction of microhardness and non-alcoholic beer had the highest effect on reducing microhardness.

## Introduction

Clinical service of restorative materials is influenced by chemical abrasion due to exposure to endogenous factors such as the gastrointestinal acids and exogenous parameters such as the acidic and alcoholic beverages.(1) Acid exposure affects the wear of composite resins.(2) Composite restorative materials might undergo destruction at the matrix/filler interface by acid attack.(3) Organic matrix of composite materials makes them more susceptible to chemical change compared with ceramic and metal restorative materials.(4) Silorane-based composite resins were recently introduced to make up for the drawback of methacrylate-based composites namely their polymerization shrinkage. Silorane-based composites undergo ring-opening polymerization through cationic mechanism.(3) The monomer of silorane-based composite is produced from the reaction of oxirane and siloxane molecules and the name is derived from the name of those two molecules. The two main advantages of these composites are low polymerization shrinkage and higher hydrophobicity.(5-9) These composite resins also benefit from less than 1.5% polymerization shrinkage,(8-9) low water sorption,(6, 10) optimal biocompatibility,(11) adequate color stability,(12) better marginal fit, and less microleakage.(13) Due to the extensive use of resin-based restorative materials and their exposure to oral environment, their clinical survival and longevity is of utmost importance. The effect of acidic and alcoholic beverages such as Coca Cola, various alcoholic beverages and juices(3, 14-16) on methacrylate-based composites has been the subject of numerous investigations. The impact of acids in the composition of these beverages on methacrylate-based composite resins has also been very well investigated.(17-18) However, studies evaluating the effect of these beverages on silorane-based composite resins are scarce.(18) On the other hand, due to the health benefits of pure juices and higher popularity of non-alcoholic beverages among the Iranian population, this study sought to assess and compare the effect of orange juice, non-alcoholic beer, and distilled water on one silorane-based and two methacrylate-based composite resins by using the microhardness test.

## Materials and Method

**Preparation of specimens**

Disc-shaped composite specimens with 2mm thickness and 10mm diameter were fabricated by using a stainless steel mold. The mold was filled with composite resin between two transparent matrix bands according to the manufacturer’s instructions. The specimens were light-cured for 20s from each side of the mold by using an LED light-curing unit (Valo; Ultradent Products Inc., South Jordan, UT, USA) with 1100 mW/cm(2) intensity. A total of 90 specimens (30 of each composite resin) were fabricated and polished by a single operator using 1200, 1500, 2000, 2500, 3000 and 5000 grit silicon carbide abrasive papers (MATADOR; Yangzhong Lifeng Emery Cloth Co. China). Polished specimens were immersed in distilled water and in an ultrasonic bath for 4 minutes. Next, the samples were immersed in distilled water at room temperature for 24 hours.

**Immersion in understudy beverages**

Each group was randomly divided into 3 subgroups (n=10). Group 1 (control) was immersed in distilled water, group 2 in 100% natural noncarbonated orange juice (Sunich; Ali Fard Co., Iran) and group 3 in non-alcoholic carbonated beer (Behnoush Co.; Iran) within opaque screw-top glass vials containing 10ml of the respective solution 3h/day. At the end of 3 hours, the specimens were rinsed under running water and cleaned with a very soft tooth brush. They were then stored in distilled water at room temperature. Distilled water was refreshed daily in all groups. By use of a digital pH-meter before immersion, the pH of solutions was measured to be around 3.7 for orange juice and 3.3 for non-alcoholic beer.

**Microhardness test**

Microhardness of samples was measured in 24h (baseline), 7 days and 28 days by using a digital microhardness tester (Vickers; KB HardWin XL, KB Pruftechnik GmbH, Germany). A 100g load was applied for 20s by the indenter of Vickers machine at room temperature. Three indentations were made on each sample with more than 1mm distance from each other at different areas of the specimen surface and the mean microhardness was calculated using the 3 obtained values. The Vickers microhardness was calculated by measuring the diagonal lengths of each indentation through the following equation(19)

HV=1.854F.d^2^

where F is the applied load and d is the average of diagonal lengths of the indentation.

**SEM analysis**

Two specimens in each group were prepared for observation in scanning electron microscope (SEM). These specimens were gold coated using a sputter coater and examined under a SEM at 20 kV voltages and 3000X magnification.

**Statistical analysis**

Data were analyzed by using repeated measures ANOVA. Microhardness at different time points was considered as the repeated factor and type of solution as the between-subjects factor. If the interaction was significant, two-way ANOVA was used for microhardness analysis at each time point and one-way ANOVA was applied to assess the effect of type of composite on microhardness in each solution as well as the effect of type of solution on microhardness of each resin. Tukey’s HSD test was used for pair-wise and multiple comparisons (α=0.05). 

## Results

**Microhardness test**

The mean microhardness values are shown in Table 1. Repeated measures ANOVA demonstrated that the interaction between the type of composite and microhardness changes was statistically significant (*p*< 0.001). Thus, two-way ANOVA was applied which revealed that at baseline, the effect of interaction of independent variables on microhardness was not significant (*p*= 0.326). The effect of type of beverage on microhardness was not significant either (*p*= 0.998). But, the effect of type of composite on microhardness was statistically significant (*p*< 0.001). Microhardness of P90 was lower than that of Z350 and the microhardness values of both were lower than that of Z250.

**Table 1:** Microhardness values (±SD)

	**Z250**			**Z350**			**P90**		
	**Baseline**	**7 days**	**28 days**	**Baseline**	**7 days**	**28 days**	**Baseline**	**7days**	**28 days**
Distilled water	91.5±5.78	81.86±4.57	72.83±3.80	80.03±30.78	76.80±2.85	72.40±3.94	73.76±7.00	63.60±4.67	62.50±3.95
100% orange juice	89.90±7.61	80.46±9.62	74.33±9.12	81.81±6033	75.81±9.22	72.77±1.51	73.50±4.02	65.53±2.35	65.36±3.51
Non- alcoholic beer	90.60±6.86	83.30±3.68	77.96±4.36	84.16±4.48	78.10±3.00	71.76±3.51	70.70±4.50	66.13±3.89	69.66±1.81

On day 7, the effect of interaction of independent variables on microhardness was not significant (*p*= 0.886). The effect of type of beverage on microhardness was not significant either (*p*= 0.328). However, the effect of type of composite on microhardness was statistically significant (*p*< 0.001). Microhardness of P90 was lower than that of Z250 and Z350 (*p*< 0.001). Microhardness value of Z350 was also lower than that of Z250 (*p*= 0.002).

On day 28, the effect of interaction of independent variables on microhardness was not significant (*p*= 0.078). Type of composite had various effects on microhardness (*p*< 0.001). Microhardness value of P90 was lower than that of Z250 and Z350 (*p*< 0.001); however, Z250 and Z359 did not significantly differ in terms of microhardness value (*p*= 0.054). The microhardness of composites was significantly affected by the type of solution (*p*= 0.005). Microhardness of composite resins in non-alcoholic beer was significantly lower than that in distilled water (*p*= 0.003). Though, the microhardness value of resins in orange juice was somewhere between the two mentioned values with no significant difference with any of them (*p*> 0.05).

**SEM results**

**Figure 1:**
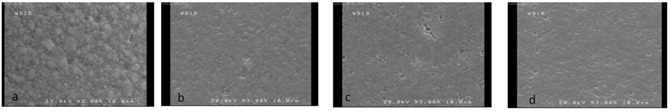
a: Z250; before immersion (left, up) b: Z250; after 28 days of immersion in distilled water (right, up) c: Z250; after 28 days of immersion in non- alcoholic beer (left, down) d: Z250; after 28 days of immersion in 100% orange juice (right, down)

**Figure 2:**
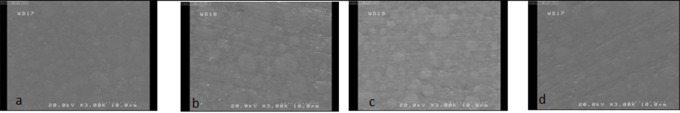
a: Z350; before immersion (left, up) b: Z350; after 28 days of immersion in distilled water (right, up) c: Z350; after 28 days of immersion in non-alcoholic beer (left, down) d: Z350; after 28 days of immersion in 100% orange juice (right, down)

**Figure 3:**
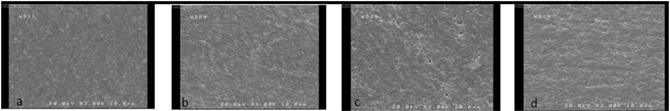
a: P90; before immersion (left, up)  b: P90; after 28 days of immersion in distilled water (right, up)  c: P90; after 28 days of immersion in non-alcoholic beer (left, down)  d: P90; after 28 days of immersion in 100% orange juice(right, down)

The images taken before and after immersion are presented in figures 1-3. After 28 days of immersion in distilled water, no significant change was perceived on composite surface. Both orange juice and non-alcoholic beer pitted the surface of P90 samples. Following immersion in the two mentioned solutions, surface of Z250 showed abrasion and wear and its surface roughness decreased but no change occurred in the surface of Z350.

## Discussion

Baseline microhardness of P90 was lower than that of methacrylate-based composite resins. Filtek P90 is filled with a combination of fine quartz and radiopaque yttrium fluoride particles and is classified as a microhybrid composite (76%). The Knoop hardness of quartz and zirconia particles is 820 and 1160, respectively.(1) Zirconia particles are incorporated in the two understudy methacrylate-based composites. On the other hand, Kusgoz *et al.*(20) demonstrated that the degree of conversion (DC) of silorane-based composites was much lower than other composites. The microhardness value depends on the DC.(21) Therefore, lower microhardness of P90 compared with the two other composites might be related to lower DC and the type of fillers. Another study indicated that silorane-based composite resins had relatively lower hardness compared with methacrylate-based composites.(9) In our study, microhardness of all understudy composite resins decreased after 28 days of immersion in orange juice and non-alcoholic beer. The acidity of orange juice is due to its citric acid content while the acidity of non-alcoholic beer is attributed to its ascorbic, citric, and lactic acid content. Citric acid is a weak carboxylic organic acid with three COOH groups found in citrus fruits (C5H6O7). The pH of non-alcoholic beer and orange juice was found to be respectively 3.3 and 3.7 in our study. The pH plays an important role in destructive effects of acidic solutions. Restorative materials are prone to be eroded under acidic conditions. The acids present in beverages penetrate into the resin matrix and cause the release of unreacted monomers, which subsequently lead to reduction of surface roughness.(14, 22-23) Abu-Bakr *et al.* reported that alcoholic beverages and soft drinks affect the compressive strength, microhardness, solubility, and surface properties of restorative materials.(14) Furthermore, de Carvalho Sales-Peres *et al.*(24) suggested that the duration of exposure to an acidic environment is much more important than the volume of consumed drink in terms of causing erosion. Thus, the erosive effects of carbonated beverages may be exaggerated because when they are consumed they are frequently held in the mouth until the bubbles gradually disappear. In the current study, microhardness reduction in samples immersed in non-alcoholic beer was greater than those in orange juice; it is probably due to the lower pH and carbonated nature of non-alcoholic beer which was refreshed daily. The fresh beverages were added in screw top vials so the CO2 bubbles remained and act efficiently to reduce the microhardness. Furthermore, organic acids present in the composition of this beverage such as lactic acid contain OH and- COOH functional groups. These functional groups are very likely to form hydrogen bonds with the polar end of methacrylate monomer present in the matrix such as- OH- in Bis-GMA, -O- in TEGDMA and Bis-EMA, and -NH- in UDMA, causing greater water sorption and consequently soften the matrix. On the other hand, low pH may affect the polymer matrix by catalyzing the ester groups. Ester groups may be hydrolyzed to alcohol and carboxylate that may accelerate the process of degradation by lowering the pH of the matrix.(25) In our study, the microhardness of P90 was lower than that of Z250 and Z350 composites and the microhardness of Z350 was lower than Z250 after 7 days of immersion in the respective solutions. According to Dos Santos *et al.*, composites with lower filler content are more wear-resistant because they are more homogenous and have less porosity and subsequently lower roughness.(26) In our study however, the final microhardness value of Filtek Z350 was lower than that of Filtek Z250. Filtek Z250 is a microhybrid composite with 0.01-3.5 μm particles. Filtek Z350 is a nanofilled composite with a filler particle system comprising of a combination of silica nanofillers with a primary size of 20nm and zirconia-silica nanoclusters sized 0.4-0.6 μm.(27) Some studies have demonstrated that this type of composite resin has mechanical properties similar to those of hybrid and midi-filled composite resins.(28-29) However, its high surface/volume ratio due to the presence of silica particles may increase its water sorption and cause destruction of polymer matrix and filler interface(30-31) compromising some of its mechanical properties.(32) Considering all the above, the understudy beverages probably affected the matrix/filler interface in this composite and caused its microhardness reduction. However, the electron microscopic images showed no surface roughness in Z350; which was probably due to the tiny filler particles in this composite and that the microhardness reduction probably occurred due to chemical softening. The electron microscopic images showed erosion of Z250 composite surface; nonetheless, its microhardness had the smallest change compared with other composites. This finding indicated that the superficial layer has undergone corrosion but less softening has occurred in the subsurface layer compared with other composites.

Excessive hydrophobicity is another characteristic of P90 and is attributed to the presence of siloxane molecule in its chemical formulation that causes its insolubility.(11) However in our study, the microhardness of P90 decreased after immersion in the solutions. Chemical softening occurs when the solubility parameter of the resin matrix of composites is similar to that of active materials.(33) No precise information is available on the solubility parameter of silorane, but the microhardness reduction in P90 was probably due to having a solubility parameter close to that of acids present in the understudy solutions. On the other hand, it has been confirmed that weak acids such as citric acid could cause degradation of inorganic fillers(34) which might play an important role in microhardness reduction.(35) The electron microscopic images demonstrated that P90 composite surface was pitted after immersion; which probably confirms the above-mentioned statement. Moreover, low pH may also be responsible for filler surface erosion and accelerated debonding of filler particles.(36)

## Conclusion

Under the limitations of this study, although 28 days of immersion in 100% orange juice and non-alcoholic beer decreased the microhardness of all specimens, P90 experienced the greatest reduction in microhardness and non-alcoholic beer had the highest effect on reducing microhardness. 
